# Dynamic Expression of BCL6 in Murine Conventional Dendritic Cells during *In Vivo* Development and Activation

**DOI:** 10.1371/journal.pone.0101208

**Published:** 2014-06-30

**Authors:** Ting-ting Zhang, Dong Liu, Samuele Calabro, Stephanie C. Eisenbarth, Giorgio Cattoretti, Ann M. Haberman

**Affiliations:** 1 Department of Laboratory Medicine, Yale School of Medicine, New Haven, Connecticut, United States of America; 2 Department of Immunobiology, Yale School of Medicine, New Haven, Connecticut, United States of America; 3 Department of Pathology, University of Milano-Bicocca, Monza (MB), Italy; Oklahoma Medical Research Foundation, United States of America

## Abstract

The transcriptional repressor BCL6 plays an essential role in the development of germinal center B cells and follicular helper T cells. However, much less is known about the expression and function of BCL6 in other cell types. Here we report that during murine dendritic cell (DC) ontogeny in vivo, BCL6 is not expressed in bone marrow hematopoietic stem cells, common DC precursors and committed precursors of conventional DCs (pre-cDCs), but is elevated in peripheral pre-cDCs. BCL6 protein levels rise as pre-cDCs differentiate into cDCs in secondary lymphoid organs. Elevated protein levels of Bcl6 are observed in all cDC subsets, with CD8α^+^ cDCs displaying the greatest levels. Co-staining of Ki-67 revealed BCL6^hi^ cDCs to be more proliferative than BCL6^lo^ cDCs. After adjuvant inoculation, BCL6 levels are significantly reduced in the CD11c^int^ MHC class II^hi^ CD86^hi^ cDCs. Activation-induced BCL6 reduction correlated with reduced proliferation. A LPS injection study further confirmed that, in response to microbial stimuli, BCL6 levels are dynamically regulated during the maturation of CD11c^int^ MHC class II^hi^ splenic cDCs. This reduction of BCL6 levels in cDCs does not occur after LPS injection in MyD88^−/−^ TRIF^−/−^ mice. Thus, regulation of Bcl6 protein levels is dynamic in murine cDCs during development, maturation and activation *in vivo*.

## Introduction

The transcriptional repressor BCL6 contains a N-terminal BTB/POZ domain and C-terminal zinc finger DNA-binding motifs and represses transcription of a wide range of target proteins and microRNAs. BCL6 is critical for the development, differentiation or function of several cell types [1]–[3]. During past decades, the roles of BCL6 in germinal center (GC) B cell development have been extensively studied [3]–[6]. Those studies indicate that BCL6 is highly expressed in GC B cells, but subject to multiple layers of regulation, the dysregulation of which can lead to lymphomagenesis [7]. Within GCs, BCL6 modulates a broad spectrum of genes related to B cell activation, tolerance of DNA damage, cell cycle arrest, plasma cell differentiation, NF-kB signaling, and apoptosis [7]. BCL6 is required for the development of T follicular helper T cells (T_FH_), a helper T cell subset required for the formation of mature and productive GCs [1], . BCL6 promotes differentiation towards the T_FH_ state by repressing Th1, Th2 or Th17 lineage-specific transcription factors as well as miRNAs that negatively regulate the chemokine receptor CXCR5 [10].

In addition to lymphocytes, BCL6 has also been shown to play important regulatory roles in macrophages [11], [12]. BCL6^−/−^ mice die young because of massive Th2-type inflammation, driven by nonlymphoid cells, including macrophages. BCL6 in macrophages selectively represses the expression of a subset of chemokines, such as MCP-1, MCP-3 and MRP-1 [12]. However, the expression and function of BCL6 has been less studied in many other cell types, such as DCs, despite their critical role as antigen presenting cells (APCs) during the initiation of adaptive immune responses.

DCs are a heterogeneous cell population, consisting of phenotypically distinguishable subtypes generated stepwise from progenitors with distinct function and tissue origin [13]. A previous study indicated that the development of splenic CD4^+^ cDCs and CD8α^+^ cDCs is diminished in BCL6^−/−^ mice [14]. LPS-stimulated BCL6^−/−^ DCs produce less IFN-γ and more IL-4, which may contribute to Th2 skewing [14]. Recent data collected by The Immunological Genome (ImmGen) Project showed that Bcl6 transcripts are upregulated during the transition from common DC progenitors (CDPs) to conventional DCs (cDCs) [15]. However, due to posttranscriptional regulation, abundance of Bcl6 transcripts does not reflect the quantity of protein [16], [17]. Therefore, it is necessary to examine BCL6 expression at the protein level in DCs under physiological settings. In this study, BCL6 protein levels were assessed in DC progenitors during DC ontogeny and in DCs at steady- state or inflammatory conditions in C57BL/6 mice. During DC development, BCL6 protein levels gradually increase as DC progenitors differentiate into cDCs. BCL6 is expressed in secondary lymphoid organ steady-state cDCs over a broad range, and protein levels are rapidly and selectively downregulated in a subpopulation of cDCs under inflammatory conditions. Finally, higher BCL6 levels correlate with greater expression of the proliferation marker Ki-67 in cDCs in the steady state and inflammatory conditions.

## Materials and Methods

### Ethics statement

All animals were housed at the Central Animal Care Facility (Yale University, New Haven) and treated in compliance with the guidelines established by the Yale University Institutional Animal Care and Use Committee (IACUC). The study was approved under protocol #11326 by the Yale IACUC.

### Mice and immunization

C57BL/6 mice were bred locally or purchased from The Jackson Laboratory. MyD88^−/−^ TRIF^−/−^ mice [18] were bred locally. 6 to 8 weeks old mice receiving intraperitoneal immunizations were injected with 2 mg Alum or 5 µg Lipopolysaccharides (LPS). Some mice were injected with 20 µl complete Freund’s adjuvant (CFA, Sigma) in the hind footpads. 1,2, 4 or 7 days after immunization, mice were euthanized according to the Yale IACUC guideline to isolate spleens or draining lymph nodes for further examination. For the flow cytometry and RT PCR analysis of germinal center (GC) B cells, LNs were obtained from mice carrying the Vh186.2 heavy chain derived from the B1-8 hybridoma specific for the hapten NP [19] 7 days after immunization in the hind footpads with NP-chicken γ globulin (NP-CGG)/CFA. For the RT PCR analysis of T follicular helper (Tfh) cells an adoptive transfer system was employed as described previously [20]. GFP+ OTII T cells were adoptively transferred, together with anti-NP specific B cells obtained from B1-8+/+ Jκ−/− mice, to recipients that were subsequently immunized i.p. with NP-OVA prepared in alum.

### Reagents

The following reagents were purchased from BD Bioscience, eBioscience or Biolegend: biotinylated–αCD11c and αSca-1; FITC–conjugated αI-A/I-E; Alexa Fluor 488–αCD3, αCD4, αCD8, αCD11b, αCD19, and αB220; PE–αCD11c, αCD115, αCD172α (SIRPα), αCD317 (PDCA-1); PerCP5.5–conjugated αTCRβ; PerCP-eFluor 710–αCD135; PE-Cy7–conjugated αCD11c, αCD86; Alexa Fluor 647–αBcl6 and mouse IgG1 isotype control; APC-eFluor 780–αCD117 (c-Kit); Brilliant Violet 421–αCD11c, αB220 and αI-A/I-E; and Cytoperm/Cytofix solution and Perm/Wash buffer. FITC–αKi-67 was from Abcam. Streptavidin-Alexa Fluor 555, Alexa488–Donkey anti-goat IgG, Prolong Gold antifade mounting medium, and LIVE/DEAD Fixable Yellow Dead cell stain were purchased from Invitrogen.

### Cell preparation

To prepare single cell suspensions, spleens or lymph nodes were cut into small fragments and digested for 30 min at 37°C with mixing in 10 ml Hank’s buffer containing collagenase (5 mg/ml; type II; Invitrogen). At the end of digestion, 200 µl of 0.5 M EDTA, pH 8.0, was added to disrupt DC-T cell conjugates and the cell suspension filtered through nylon mesh to remove undigested fibrous material. Bone marrow (BM) cells were harvested by injecting Hank’s medium through the end of bone. Peripheral blood cells were collected into heparin-coated tubes by cardiac puncture. Red blood cells were lysed by ACK lysis buffer (BioSource).

### Flow cytometry

Single cell suspensions were pre-incubated with Fc blocking antibody (mAb 2.4G2) and stained with LIVE/DEAD Fixable Yellow Dye to discriminate dead cells. After cell surface staining, cells were treated with Cytofix/Cytoperm solution (BD Bioscience) for 30 min at 4°C and then washed with Permeablization Buffer (BD Bioscience). Cells were incubated with 10% rat serum to block non-specific signals prior to staining with antibodies (Ab) recognizing nuclear anigen (Ag) BCL6 and/or Ki-67 overnight at 4°C. Flow cytometry was performed on a LSRII cytometer (Becton Dickinson) and analyzed with FlowJo software (TreeStar, Portland, OR). Cell populations were gated as follows: BM hematopoietic stem cells as Lin^−^ (CD3^−^ CD4^−^ CD8^−^ CD19^−^ B220^−^ CD11b^−^) CD11c^−^ Sca-1^+^c-Kit^+^; BM common pDC and cDC progenitors (CDPs) as Lin^−^ CD11c^−^ Sca-1^−^ Flt3^+^ c-Kit^l^°CD115^+^; BM, blood, lymph node and spleen pre-cDCs as Lin^−^ (CD3^−^ CD19^−^ B220^−^) CD11c^int^ I-A^−^ Flt3^+^ SIRPα^int^; Spleen and lymph node cDCs as TCRβ^−^ CD19^−^ CD11c^+^ I-A^+^; and Spleen and lymph node pDCs as TCRβ^−^ CD19^−^ CD11c^int^ I-A^−^ PCDA^+^.

### Immunofluorescence

Partial spleens were fixed in vitro with 1% paraformaldehyde-lysine-periodate solution, and frozen in OCT (TissueTek) after passage through sucrose gradient solutions. Seven µm- thick cryostat sections were blocked with 10% rat serum in PBS containing 1% bovine serum albumin (BSA) for 30 min at RT. After blocking, sections were stained with antibody reagents recognizing CD11c, MOMA-1 (CD169) or B220 in PBS containing 1% BSA for 2 h at RT. Subsequently, slides were washed and stained overnight at 4°C for the presence of BCL6. After extensive washing, the slides were mounted in Prolong Gold anti-fade reagent. Images were taken with an automated wide-field microscope (Nikon Eclipse Ti) and a CCD camera (Qimaging Retiga 2000R) with NIS Elements software.

### Real-time PCR

BM pre-cDCs, spleen pre-cDCs, cDCs, naïve B cells from naïve C57BL/6 mice, GC B cells (B220^+^Fas^+^CD38^low^) collected 7 days post-immunization and T follicular helper (T_FH_) (CD4^+^ICOS^+^CXCR5^+^) cells collected 11 days post-immunization, were sorted with a BD FACSAria. RNA from sorted cell populations was isolated using the MicroRNeasy Kit (Qiagen) followed by reverse transcription to cDNA using iScript (Bio-Rad). Quantitative PCR was performed on a Stratagene MX4005P Thermal Cycler using KAPA SYBR Fast qPCR Master Mix. BCL6 primer sequences were described in a previous publication [21]. β-actin was used as relative expression control to normalize sample variation.

## Results

### BCL6 levels are elevated in peripheral pre-cDCs during DC ontogeny in vivo

**Figure 1:**
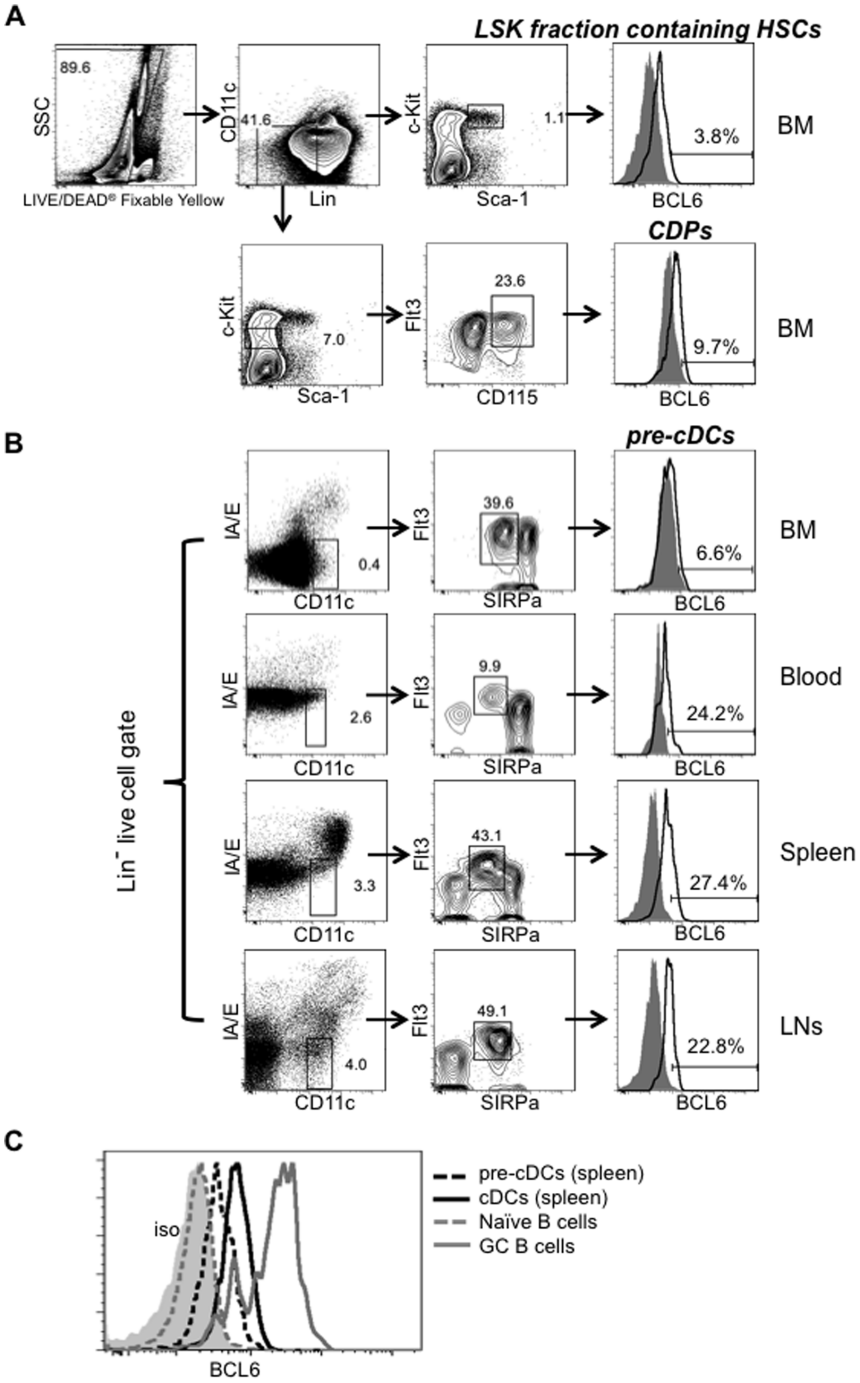
BCL6 protein is weakly expressed in peripheral pre-cDCs during in vivo development. BM, peripheral blood, spleens and LNs were collected from naïve C57BL/6 mice. *(A)* BM HSCs and CDPs were gated as indicated. The Lin markers include CD3, CD4, CD8, CD19, B220 and CD11b. Representative FACS data were shown (n = 8). *(B)* Pre-cDCs were gated as indicated usingthe Lin markers including CD3, CD19 and B220. Representative FACS plots were shown (n = 6). Mouse IgG1 isotype control (solid grey), BCL6 expression in indicated DC population (open black). BCL6 expression gates were drawn based on the negative expression of isotype controls. Numbers in the FACS plots indicated the percent of population. *(C)* Overlay of BCL6 expression levels in different cell population. BCL6 expression in GC B cells obtained 7 days p.i. (gated as B220^+^ Fas^+^ CD38^lo^ cells) served as positive controls (open grey line) and naïve B cells (gated as B220^+^ Fas^−^ CD38^hi^ cells) as negative controls (open grey dashed line).

DCs derive from BM hematopoietic stem cells (HSCs) in a stepwise manner. The earliest commitment to the DC lineage occurs during the transition from the macrophage and DC precursor (MDP) to the common DC precursor (CDP), which will give rise exclusively to pDCs and pre-cDCs [22], [23]. Pre-cDCs, expand in the BM, and travel through the blood circulation to peripheral lymphoid organs, where they further differentiate into cDCs [24]. We examined BCL6 levels in Lin^−^ Sca^+^ Kit^+^ (LSK) fraction containing HSCs, CDPs and pre-cDCs during DC development via flow cytometry. As shown in Fig. 1A, BCL6 was not expressed in the LSK fraction containing HSCs (Lin^−^ CD11c^−^ Sca-1^+^ c-Kit^+^) and CDPs (Lin^−^ CD11c^−^ Sca-1^−^ Flt3^+^ c-Kit^l^°CD115^+^) in BM. BCL6 was also not detected in BM pre-cDCs (CD3^−^ CD19^−^ B220^−^ I-A^−^ CD11c^int^ Flt3^+^ SIRPα^int^) (Fig. 1B). However, we found that BCL6 levels were moderately increased in pre-cDCs residing in blood and peripheral lymphoid organs (Fig. 1B). BCL6 expression levels in splenic pre-cDCs are lower than those observed in GC B cells and splenic cDCs (Fig. 1C). Together, these expression patterns suggest that BCL6 is not required for the transition from CDPs to pre-cDCs, or for the expansion of pre-cDCs in the BM, but is commensurate with the differentiation of pre-cDCs towards cDCs in peripheral lymphoid organs.

### Elevated BCL6 expression in cDCs under steady state is correlated with the proliferation marker Ki-67

**Figure 2:**
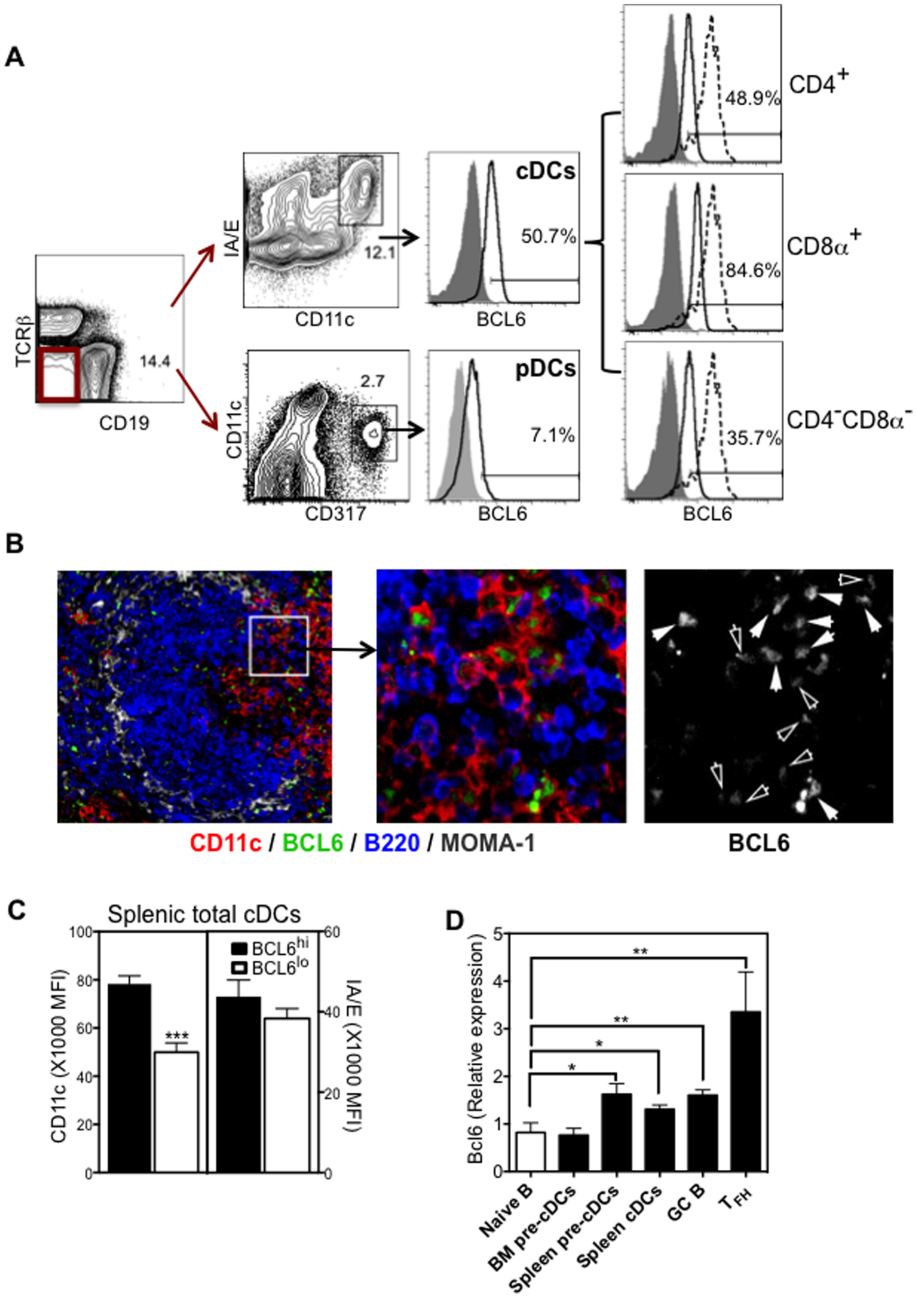
BCL6 is elevated in steady-state cDCs. *(A)* Splenocytes from naïve C57BL/6 mice were stained for cDCs subsets or pDCs, and for intracellular BCL6 protein expression. Representative FACS plots were shown (n = 8): Isotype controls (solid grey), BCL6 expression of GC B cells gated as described in Figure 1 as positive controls (dashed line), BCL6 expression of the indicated DC population (open black). BCL6 expression gates were drawn based on the negative expression of isotype controls, and the percentage of BCL6^+^ cells of the gated cDC population indicated. *(B)* Representative immunofluorescence picture shows the staining pattern of CD11c (red), BCL6 (green), B220 (blue), and MOMA-1 (grey). Solid white arrows identify CD11c^+^ cells expressing BCL6 with higher intensity, and open white arrows indentify CD11c^+^ cells expressing BCL6 with lower intensity. *(C)* Mean fluorescence intensity (MFI)±SEM (n = 4) of CD11c (left panel) and I-A/E (right panel) in either BCL6^hi^ (solid bar) or BCL6^lo^ (open bar) cDCs. *(D)* BM and splenic pre-cDCs, spleen cDCs, and naïve B cells were sorted from naïve C57BL/6 mice; control GC B cells and T follicular helper (T_FH_) cells were sorted from immunized mice as described in the methods section. Bcl6 transcript levels were determined by quantitative RT-PCR. Normalized expression is relative to the corresponding β-actin control. Bar Graph shows the relative expression ± SEM (n = 3) of Bcl6 in different DC populations and control cells. *p<0.05; **p<0.005; ***p<0.001 (students T-test) as compared to Bcl6 transcripts in Naïve B cells.

It has been previously reported that cDCs, but not pDCs, have abundant Bcl6 mRNA transcripts [14], [15]. Consistent with this, we observed a broad range of BCL6 expression within the entire splenic cDC population (Fig. 2A). The expression level of BCL6 in cDCs was lower than that of germinal center B cells (Fig. 1C). Subsets of splenic cDCs differed in the expression levels of BCL6, with CD8α^+^ cDCs displaying the greatest levels, and CD4^−^ CD8α^−^ cDCs the lowest (Fig. 2A). Negligible expression of BCL6 was detected in pDCs (Fig. 2A), consistent with a previous finding indicating that BCL6 is not required for pDC development and function [14]. Immunofluorescence histology of spleens obtained from naïve mice clearly demonstrates the presence of nuclear BCL6 in some CD11c^+^ DCs located in both marginal zones and periarterial lymphatic sheaths (PALS) (Fig. 2B). DCs expressed BCL6 at different levels with the majority showing low to intermediate intensity (indicated by open arrows in figure 2B), and others with high intensity (indicated by solid arrows in figure 2B). Within splenic CD11c^+^ and I-A/E^+^ total cDCs, flow cytometry analysis suggested a positive correlation of BCL6 expression with CD11c, but not with MHC class II molecules. As quantified by the mean fluorescence intensity (MFI), expression levels of CD11c in BCL6^hi^ cDCs were higher than that in BCL6^lo^ cDCs, whereas there was no significant difference in the expression of I-A/E between BCL6^hi^ and BCL6^lo^ cDCs (Fig. 2C). Consistent with our findings of BCL6 protein expression in DC populations, quantitative RT-PCR results indicate that Bcl6 transcripts are elevated in splenic pre-cDCs and cDCs, but not in BM pre-cDCs (Fig. 2D).

**Figure 3:**
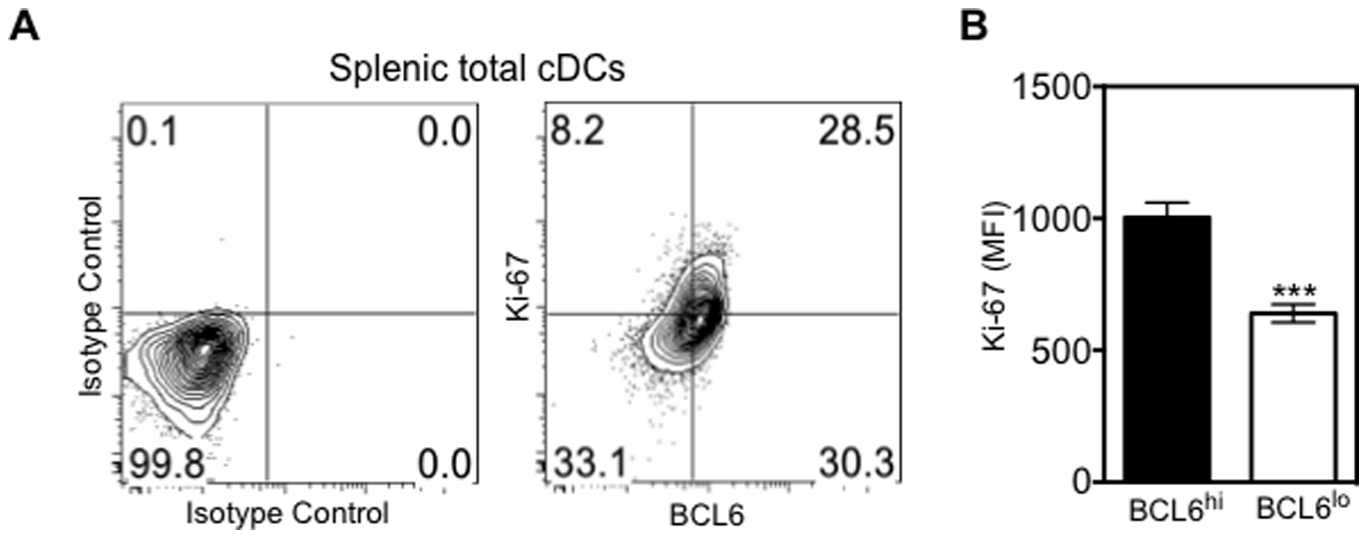
BCL6 protein levels correlates with the proliferation marker Ki-67. Splenic cDCs were further analyzed for intracellular BCL6 and Ki-67 expression. *(A)* Co-expression of BCL6 and Ki-67 in splenic total cDCs (right plot). Expression was gated based on staining of mouse IgG1 and Rabbit IgG isotype controls (left plot). *(B)* Bar Graph shows the MFI of Ki-67± SEM (n = 4) in BCL6^hi^ (solid bar) or BCL6^lo^ (open bar) cDCs. **p<0.005 (Students T-test).

In GC B cells, BCL6 has been shown to promote cell proliferation by suppressing cell cycle arrest [6]. cDCs in peripheral lymphoid organs are short-lived, but are able to proliferate and undergo limited rounds of cell divisions [25]. To assess whether BCL6 is associated with proliferation in cDCs, we stained cells with both BCL6 and nuclear proliferation marker Ki-67. As shown in Figure 3, the MFI of Ki-67among BCL6^hi^ cDCs was approximately two times higher than that in BCL6^lo^ cells, indicating that BCL6^hi^ cDCs are more proliferative. Together, our flow cytometry and immunofluorescence data suggest that steady-state cDCs, but not pDCs, express BCL6 protein and that expression of BCL6 correlates with proliferation.

### BCL6 protein levels are rapidly reduced in the CD11c^int^ I-A^hi^ subpopulation of secondary lymphoid organ cDCs after adjuvant inoculation

**Figure 4:**
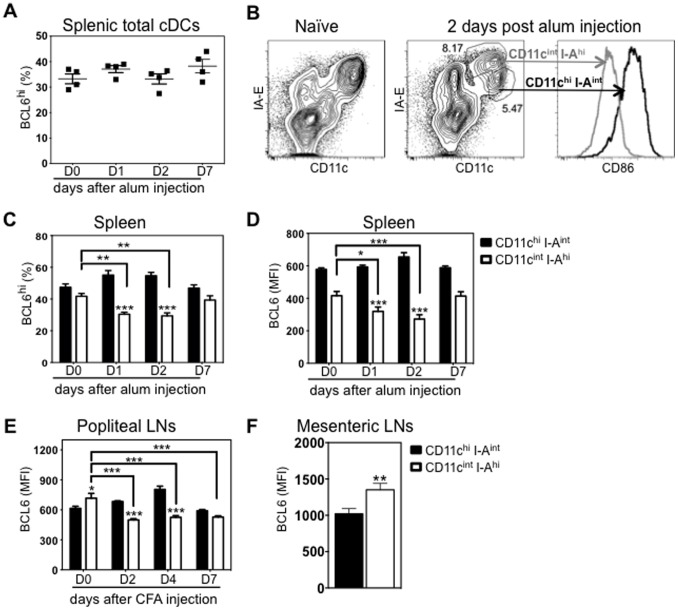
BCL6 is transiently downregulated in CD11c^int^ I-A^hi^ subpopulation of cDCs in secondary lymphoid organs after Alum or CFA injection. C57BL/6 mice were immunized with Alum by intraperitoneal injection or with CFA by footpad injection. *(A)* The average percent ±SEM (n = 4) BCL6^hi^ in splenic total cDCs (CD11c^+^ I-A^+^) before and after Alum injection. *(B)* By D2 after immunization, representative FACS data shows splenic CD11c^hi^ I-A^int^ and CD11c^int^I-A^hi^ subpopulations (gated on single live CD19^−^ TCRβ^−^ cells), which are absent in unimmunized mice, and their CD86 expression (n = 4). CD11c^hi^ I-A^int^ (grey line) and CD11c^int^ I-A^hi^ (black line). *(C)* The average percent ±SEM (n = 4) BCL6^hi^ in splenic cDC subpopulations before and after alum injection. *(D)* The average BCL6 MFI ±SEM (n = 4) in splenic cDC subpopulations before and after alum injection. *(E)* The average BCL6 MFI ±SEM (n = 4) in LN cDC subpopulations before and after CFA footpad injection. *(F)* The average BCL6 MFI ±SEM (n = 4) in cDC subpopulations of mesenteric LNs under steady state. CD11c^hi^I-A^int^ (solid bar) and CD11c^int^I-A^hi^ (open bar); One way and two way ANOVA multiple comparasions were used (within and between cDC subpopulation), *p<0.05; **p<0.005; ***p<0.001 (ANOVA) was compared either as indicated or with their counterparts.

DCs play a critical role in sensing environmental cues to initiate appropriate adaptive immune responses [26]. To examine whether modulation of BCL6 levels in DCs occurs during this process, we immunized C57BL/6 mice by intraperitoneal injection of Alum, and then examined BCL6 levels in splenic cDCs at day 1, 2, 4 and 7 after immunization. Alum has been widely used as an adjuvant to boost immune responses, but the nature of its activition of DCs is poorly understood. Previous studies indicate that alum may activate DCs via inflammasome pathway [27], and/or in part promote DC maturation and antigen presentation by precipitating host DNA [28]. Percentages of BCL6^hi^ cells in CD11c^+^ I-A^+^ cDCs were comparable before and after Alum immunization (Fig. 4A). However, one to two days after Alum immunization, splenic cDCs phenotypically defined by CD11c and I-A expression were more heterogeneous with a more pronounced CD11c^int^ I-A^hi^ subpopulation (Fig. 4B). Splenic CD11c^int^ I-A^hi^ cDCs expressed higher levels of costimulatory molecule CD86 than CD11c^hi^ I-A^int^ cDCs (Fig. 4B), consistent with the idea that CD11c^int^ I-A^hi^ cDCs are an in vivo activated subpopulation. Within the CD11c^hi^ I-A^int^ subpopulation, BCL6 expression levels (analyzed either by the percentage of BCL6^hi^ or BCL6 MFI) were unchanged after Alum injection (Fig. 4C and D). By contrast, within the CD11c^int^ I-A^hi^ subpopulation, the percentage of BCL6^hi^ cells or BCL6 MFI was significantly reduced one and two days post immunization, but returned to comparable levels seven days post immunization (Fig. 4C and D). The percentage of BCL6^hi^ or BCL6 MFI in CD11c^int^ I-A^hi^ cells was lower than that in CD11c^hi^ I-A^int^ cells, a difference that was more striking one and two days post immunization when in vivo maturation/activation was most evident (Fig. 4C and D).

Lymph node (LN) cDCs are more heterogeneous than splenic cDCs, including both resident cDCs and peripheral tissue migrating cDCs under inflammatory and steady-state conditions [29]. In the steady state, resident and migratory cDCs can be distinguished by their expression levels of CD11c and I-A, CD11c^hi^ I-A^int^ or CD11c^int^ I-A^hi^ respectively [30]. In an inflammatory setting, the CD11c^int^ I-A^hi^ subpopulation contains both migratory cDCs and activated resident cDCs [29]. To address whether BCL6 levels are also modulated during inflammation, we inoculated C57BL/6 mice with complete Freud’s adjuvant (CFA) by footpad injection and assessed BCL6 levels in popliteal LN cDCs. Similar to splenic cDCs, the BCL6 MFI in CD11c^hi^ I-A^int^ LN cDCs was not reduced after CFA injection (Fig. 4E). Within LN CD11c^int^ I-A^hi^ subpopulation, BCL6 MFI was markedly reduced two days after CFA footpad injection, and continued to remain at this lower level over time (Fig. 4E). Collectively, we found that in vivo activation of splenic or LN cDCs by means of injection with Alum or CFA leads to a rapid reduction of BCL6 expression in the CD11c^int^ I-A^hi^ subpopulation. To be noted, in the steady state, the MFI of BCL6 in LN CD11c^int^ I-A^hi^ cDCs (migratory cDCs) was even higher than that in CD11c^hi^ I-A^int^ cDCs (resident cDCs) (Fig. 4E). A higher BCL6 MFI in steady-state migratory cDCs was also observed in mesenteric LNs (Fig. 4F).

### Activation-induced BCL6 downregulation is accompanied by reduced proliferation

**Figure 5:**
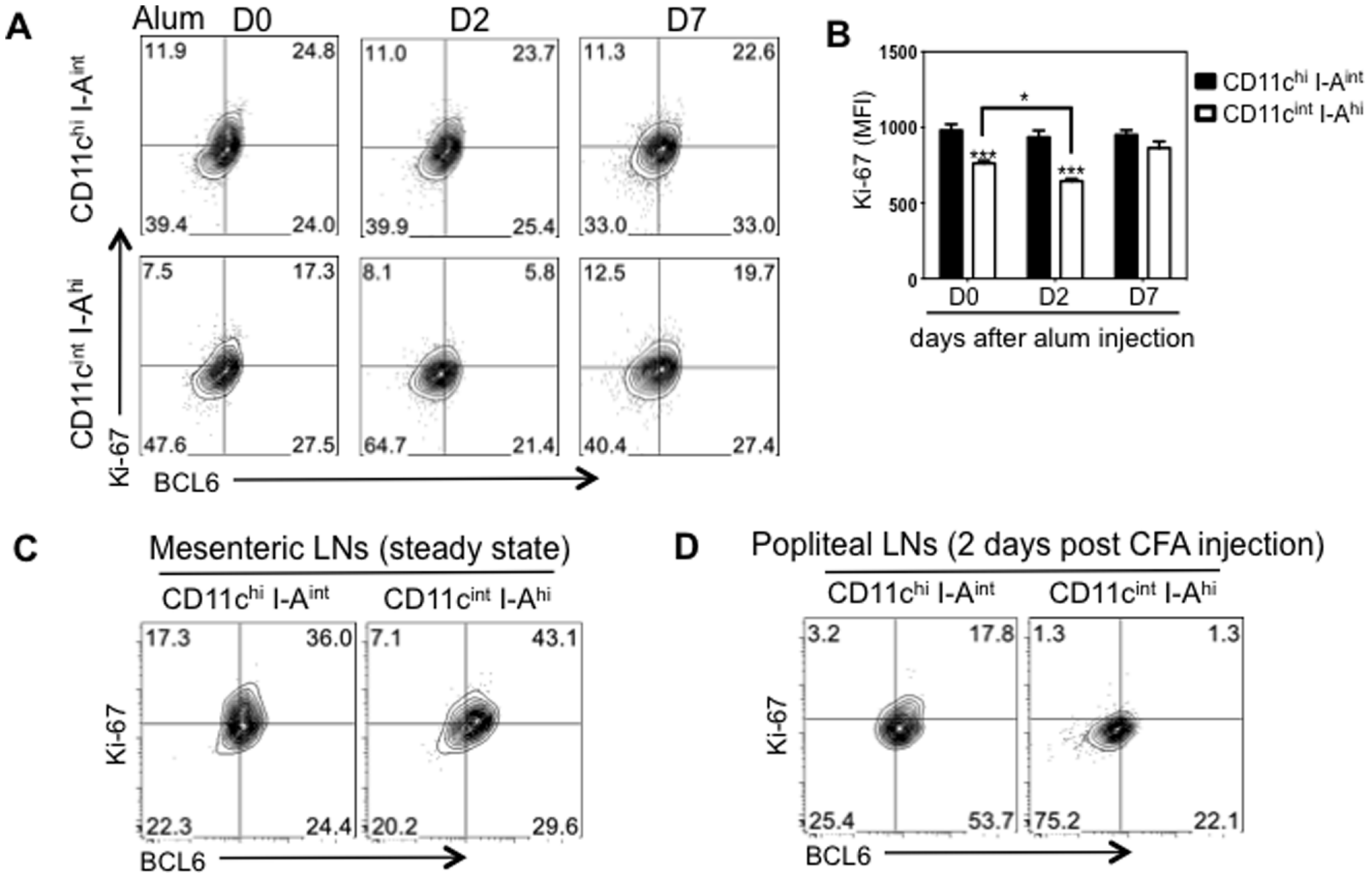
BCL6 downregulation in CD11c^int^ I-A^hi^ cells is correlated to reduced proliferation. Splenocytes or LN cells obtained before and after Alum/CFA inoculation were stained with BCL6, Ki-67 and cDC markers. *(A)* Representative FACS plots (n = 4) showing Ki-67 and BCL6 expression on gated either CD11c^hi^ I-A^int^ or CD11c^int^ I-A^hi^ splenic cDCs at day 0, 2 and 7 after Alum i.p. *(B)* The average MFI of Ki-67±SEM (n = 4) in either CD11c^hi^ I-A^int^ (solid black bar) or CD11c^int^ I-A^hi^ (open bar) splenic cDCs. *(C)* and *(D)* Representative flow cytometry data (n = 4) showing Ki-67 and BCL6 expression in CD11c^hi^ I-A^int^ or CD11c^int^ I-A^hi^ cells of mesenteric LNs under steady state *(C)* or of draining popliteal LNs two days post CFA injection *(D)*. *p<0.05, ***p<0.001 (Two way ANOVA) was compared either as indicated or with its counterpart.

To investigate whether lowered BCL6 levels in CD11c^int^ I-A^hi^ cDCs is associated with a change in proliferation, expression of the proliferation marker Ki-67 in CD11c^hi^ I-A^int^ and CD11c^int^ I-A^hi^ cDCs was assessed. The Ki-67 levels of gated CD11c^hi^ I-A^int^ cDCs were comparable after injection of Alum, consistent with their unaltered expression of BCL6 (Fig. 4A and B). In line with the significantly reduced BCL6 expression by splenic CD11c^int^ I-A^hi^ cDCs two days after Alum i.p. injection, the MFI of Ki-67 in this subpopulation also declined (Fig. 5A and B). Concurrently, splenic CD11c^int^ I-A^hi^ cDCs were much less proliferative compared to CD11c^hi^ I-A^int^ cDCs (Fig. 5A and B). In LNs, the association between BCL6 and Ki-67 was also detected (Fig. 5C and D). Under steady state conditions, migratory cDCs in mesenteric LNs expressed high levels of BCL6, retaining a proliferative capacity comparable to resident cDCs (Fig. 5C). Upon local inflammation, two days after CFA footpad injection when BCL6 was greatly reduced, draining LN CD11c^int^ I-A^hi^ cDCs displayed diminished Ki-67 levels relative to CD11c^hi^ I-A^int^ cDCs (Fig. 5D). All together, our results indicate that BCL6 expression levels in cDCs correlates with their proliferative status.

### In vivo LPS stimulation dynamically affects BCL6 expression, preferentially in CD8α^+^ splenic cDCs

**Figure 6:**
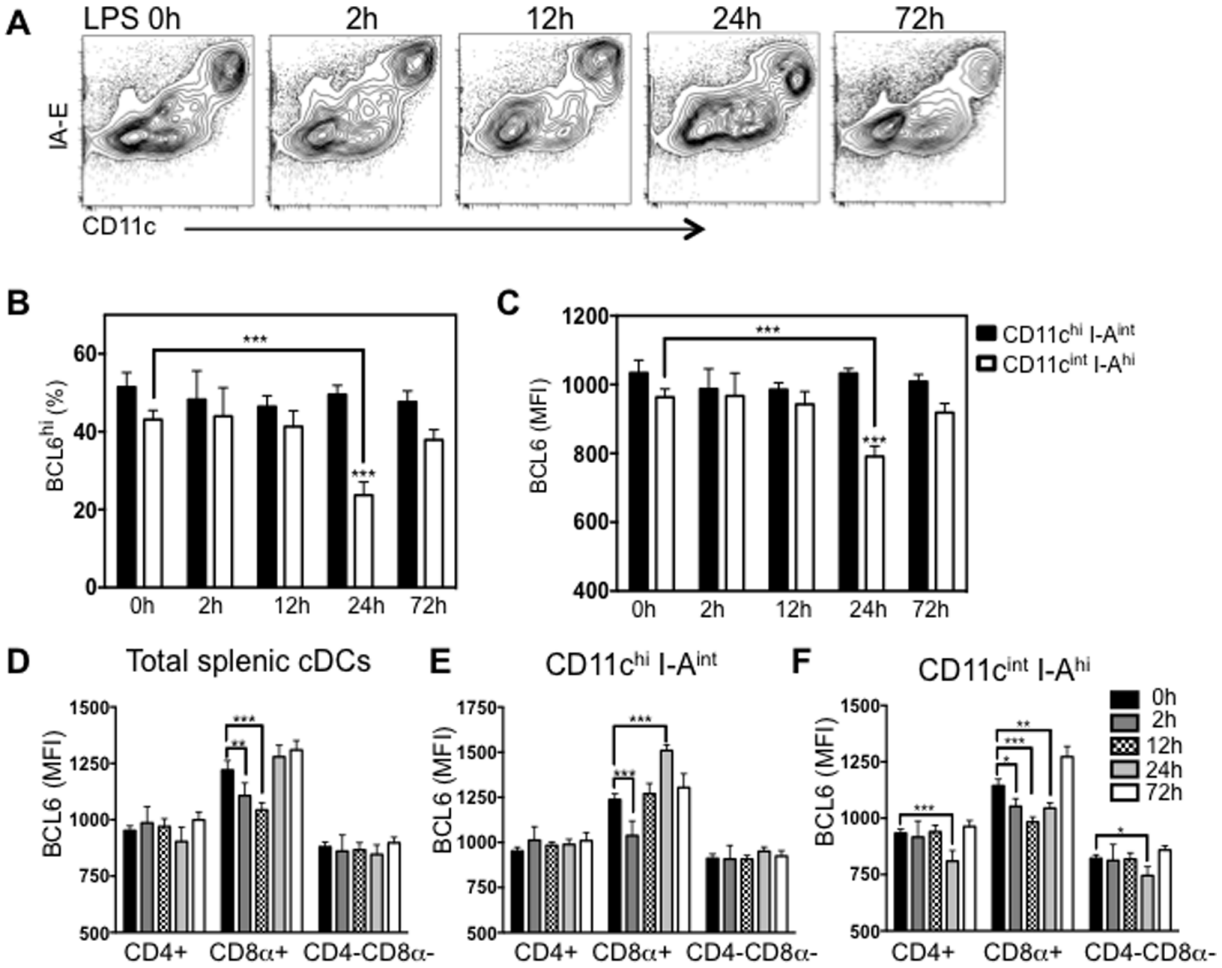
LPS injection dynamically affects BCL6 expression, preferentially in splenic CD8α^+^ cDCs. C57BL/6 mice were injected with TLR4 agonist LPS by *i.p.* route. *(A)* After LPS injection, representative FACS data (n = 4) showing transient segregation of two subpopulations of total cDCs. *(B)* The average percent ±SEM (n = 4) BCL6^hi^ in splenic CD11c^hi^ I-A^int^ (balck solid bar) or CD11c^int^ I-A^hi^ (black open bar) cDCs before and after LPS injection. *(C)* The average BCL6 MFI ±SEM (n = 4) in splenic CD11c^hi^ I-A^int^ (balck solid bar) or CD11c^int^ I-A^hi^ (black open bar) cDCs before and after LPS injection. *(D)* Bar graph showing the average MFI ±SEM (n = 4) of BCL6 in cDC subsets (CD4^+^, CD8α^+^ and CD4^−^CD8α^−^) of total splenic cDCs at 0 h, 2 h, 12 h, 24 h and 72 h after LPS injection. *(E)* and *(F)* The average MFI ±SEM (n = 4) of BCL6 in CD4^+^, CD8α^+^ and CD4^−^CD8α^−^ subsets within either CD11c^hi^ I-A^int^ *(C)* or CD11c^int^ I-A^hi^ *(D)* cells at indicated hours after LPS injection. *p<0.05; **p<0.005; ***p<0.001 (two way ANOVA) with multiple comparisons was made between groups *(B and C)* or within each group compared to values at 0 h *(B, C, D, E and F)*.

In human DCs, maturation signals induce transcriptional downregulation of BCL6 [31]. To determine whether reduced BCL6 levels in murine CD11c^int^ I-A^hi^ cDCs is also observed during a response to microbial stimuli, mice were injected with LPS i.p. and BCL6 levels examined in splenic cDC subsets 2 h, 12 h, 24 h or 72 h later. Two hours After LPS immunization, we found an increase of I-A expression within the total cDC population, which became more evident at 12 h after LPS immunization (Fig. 6A). 72 h after LPS immunization, CD11c^int^ I-A^hi^ subpopulation had declined, leaving a more homogeneous population, comparable to splenic cDCs in naïve mice (Fig. 6A). Protein levels of BCL6 in CD11c^int^ I-A^hi^ cDCs were markedly reduced by 24 h post LPS injection (Fig. 6B and C).

When total cDCs were further subdivided into CD4+, CD8α+, or CD4-CD8α- cDC subsets, we observed significant reduction of BCL6 expression only in CD8α+ cDCs at 2 h and 12 h after LPS injection, but not in CD4+ cDCs and CD4-CD8α- cDCs (Fig. 6D). Interestingly, 12 h after LPS injection, we found that CD8α+ cDCs mostly had a CD11cint I-Ahi phenotype (data not shown). Within CD11chi I-Aint subpopulation, BCL6 in CD4+ cDCs and CD4-CD8α- cDCs remained at comparable levels before and after LPS injection (Fig. 6E). Reduced BCL6 expression in CD8α+ cDCs was observed two hours after LPS injection (Fig. 6E). Of note, 24 h after LPS injection, expression levels of BCL6 in CD8α+ cDCs were even higher than their basal levels before injection (Fig. 6E). Within CD11cint I-Ahi subpopulation, BCL6 in CD8α+ cDCs was rapidly downregulated as early as 2 h. By 12 h after LPS injection, BCL6 expression in CD8α+ cDCs reached to the lowest level (Fig. 6F). At 72h, BCL6 in CD8α+ cDCs was fully recovered to the high expression levels observed prior to LPS injection (Fig. 6F). In contrast, a dynamic fluctuation of BCL6 expression in CD4+ cDCs and CD4-CD8α- cDCs was less evident (Fig. 6F). Taken together, BCL6 levels in splenic CD11cint I-Ahi cDCs is rapidly decreased after LPS injection. Moreover, BCL6 expression in CD8α+ cDCs is subjected to more dynamic modulation in vivo upon LPS stimulation.

### LPS-induced reduction of BCL6 levels in cDCs does not occur in MyD88^−/−^ TRIF^−/−^ mice

**Figure 7:**
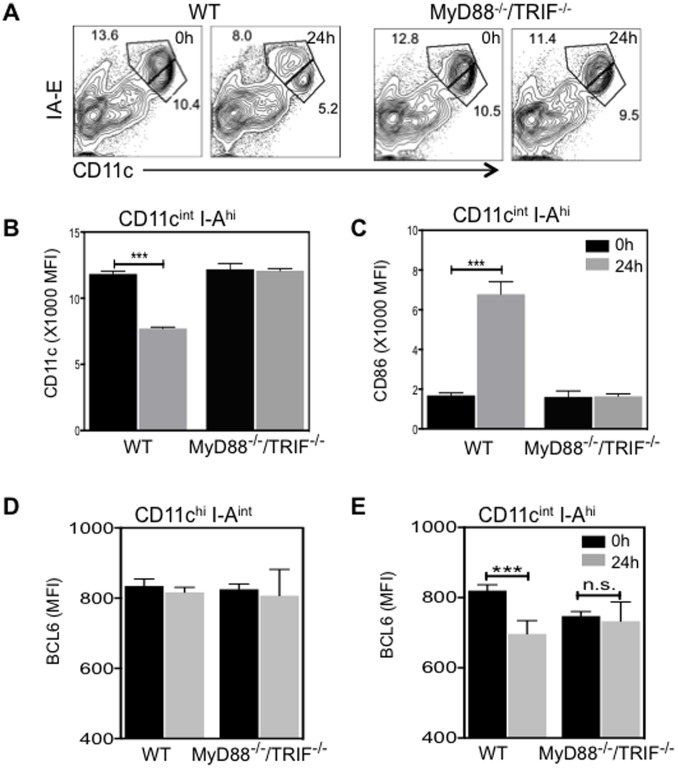
LPS-induced rapid reduction of BCL6 levels does not occur in MyD88^−/−^ TRIF^−/−^ mice. WT or MyD88^−/−^ TRIF^−/−^ mice were *i.p.* injected with LPS. *(A)* Representative FACS plots (n = 4) showing CD11c and I-A/E expression on splenic cells in 0 h or 24 h post-LPS injected mice. *(B)* and *(C)* The MFI±SEM (n = 4) of CD11c *(B)* and CD86 *(C)* in gated splenic CD11c^int^ I-A^hi^ cells obtained from either WT or MyD88^−/−^ TRIF^−/−^ mice 0 h (black bar) or 24 h after (grey bar) LPS injection. *(D)* and *(E)* The average MFI ±SEM (n = 4) of BCL6 in CD11c^hi^ I-A^int^ *(D)* and CD11c^int^ I-A^hi^ *(E)* cells 0 h (black bar) and 24 h after (grey bar) LPS injection. ***p<0.001 (two way ANOVA) was compared within each group.

LPS stimulates DCs by binding to the cell surface Toll-like receptor 4 (TLR4), which leads to recruitment of adaptor molecules MyD88 and TIR-domain-containing adaptor-inducing interferon (TRIF). MyD88 and TRIF further recruit downstream kinases to transmit signals for the induction of active NF-κB and of inflammatory cytokines [32]. To examine whether LPS-induced decrease of BCL6 in CD11c^int^ I-A^hi^ cDCs is dependent upon signaling via the adaptor molecules MyD88 and TRIF, MyD88^−/−^ TRIF^−/−^ mice were i.p. injected with 5 µg LPS. At 24 h after LPS injection, we found that separation of the two subpopulations observed in WT splenic cDCs was absent in MyD88^−/−^ TRIF^−/−^ splenic cDCs (Fig. 7A). In WT CD11c^int^ I-A^hi^ cells, CD11c was significantly decreased and CD86 expression was markedly upregulated at 24 h post LPS injection (Fig. 7B and C). However, in the absence of MyD88 and TRIF, no changes in the expression of CD11c and CD86 were observed in gated CD11c^int^ I-A^hi^ cells after LPS injection (Fig. 7B and C). Furthermore, we examined BCL6 expression in subpopulations of cDCs from either WT or MyD88^−/−^ TRIF^−/−^ mice. Expression levels of BCL6 in WT and MyD88^−/−^ TRIF^−/−^ CD11c^hi^ I-A^int^ cDCs were comparable under steady state conditions and remained unchanged after LPS treatment (Fig. 7D). In contrast, expression levels of BCL6 in WT CD11c^int^ I-A^hi^ cDCs were significantly reduced, but remained at comparable levels in the CD11c^int^ I-A^hi^ cDCs of MyD88^−/−^ TRIF^−/−^ mice at 24 h after LPS injection (Fig. 7E). Moreover, the marked reduction of BCL6 expression in CD8α+ subset of CD11c^int^ I-A^hi^ cDCs observed in WT mice was absent in MyD88^−/−^ TRIF^−/−^ mice (data not shown). Thus, in vivo LPS-induced formation of a pronounced CD11c^int^ I-A^hi^ subpopulation with lowered BCL6 levels does not occur in MyD88^−/−^ TRIF^−/−^ mice.

## Discussion

The results presented in this study indicate that during DC development *in vivo*, BCL6 protein levels gradually increase in cDC-committed precursor pre-DCs, but reach higher levels in terminally differentiated CD4^+^, CD8^+^ and CD4^−^ CD8^−^ splenic cDCs, with the most intense expression in CD8^+^ cDCs. In contrast, BCL6 protein is not expressed in Lin- Sca+ Kit+ (LSK) fraction containing HSCs, CDPs, and pDCs. Our results are consistent with previous studies of BCL6 transcripts in DCs, and the observation that cDCs numbers are diminished in BCL6^−/−^ mice, but pDCs development appears unaffected [14], [15]. It has been demonstrated that cDCs, but not pDCs, continue to divide in situ in peripheral lymphoid organs [25]. Here, we report that BCL6^hi^ cDCs are more proliferative. BCL6 is known to repress transcription of cyclin-dependent kinase inhibitors, allowing for progression through some cell cycle checkpoints [6]. Therefore, it is tempting to speculate that, under steady state conditions, regulation of BCL6 in cDCs may play a role in cDC homeostasis.

In the absence of inflammatory stimuli, BCL6 protein levels vary between cDC subsets. After inoculation with either adjuvants or the TLR4 ligand LPS, BCL6 is rapidly and transiently modulated in a subpopulation of splenic cDCs. Intraperitoneal injection of stimuli led to the emergence of a transient subpopulation of splenic cDCs that were CD11c^int^ I-A^hi^ and expressed higher levels of the co-stimulatory molecules CD86 and CD80 than CD11c^hi^ I-A^int^ cells, while CD11c^hi^ I-A^int^ cells express levels of CD86 and CD80 comparable to cDCs before immunization. Ly6C+ CD14+ cells (monocyte-derived DCs) are infrequent among Alum/LPS-induced CD11c^int^ I-A^hi^ cells (Fig. S1) supporting the idea that, after alum/LPS injection, CD11c^int^ I-A^hi^ cells may represent splenic cDCs that have matured in situ from the immature CD11c^hi^ I-A^int^ subpopulation. It is tempting to speculate that BCL6 downregulation in activated cDCs may result in derepression of BCL6 target genes, such as NFκB and CD80 [11], [33]. permitting cDC maturation and accessory cell function during adaptive immune responses. We found that CD11c^int^ I-A^hi^ cells are less proliferative than CD11c^hi^ I-A^int^ cells, consistent with the idea that a decline of BCL6 mediated repression may limit the expansion of activated cDCs and the duration of the inductive phase of adaptive immune responses.

Lymph node cDCs are more heterogeneous than splenic cDCs, consisting of not only lymphoid tissue-resident cDC subsets but also migrated cDC subsets derived from non-lymphoid tissues. Under steady state conditions, skin-draining LN and mesenteric LN migratory cDCs (CD11c^int^ I-A^hi^) express higher levels of BCL6 protein than resident cDCs (CD11c^hi^ I-A^int^). Following immunization, BCL6 levels are transiently reduced within the LN CD11c^int^ I-A^hi^ cDC subpopulation, including migratory cDCs and activated resident cDCs that serve as key players in the initiation of adaptive immune response. Steady-state migratory cDCs has been implicated in the induction of self-tolerance [34]. Recent data from the ImmGen Project indicates that activated cDCs have higher transcript levels of inflammatory cytokines compared to steady-state migratory cDCs [15]. Here we report that BCL6 protein levels are high in steady-state migratory cDCs, but low in CD11c^int^ I-A^hi^ cDCs after exposure to LPS or adjuvants, suggesting that the transcriptional repressor BCL6 might play a role in the induction of immune tolerance of self-antigens in the steady state. In line with this idea, the most intense BCL6 expression is observed in the steady-state splenic CD8α^+^ and LN CD103^+^ cDC subpopulations (data not shown), subsets reported to cross-present self-antigens from dead cells to induce self-tolerance [35], [36].

The results presented here indicate that the reduction of BCL6 levels in response to LPS stimulation in vivo is more extensive within the CD8α^+^ splenic cDC subset. BCL6 protein levels were reduced in CD8α^+^ splenic cDCs as early as 2 hr after LPS injection, and then maintained at lower levels in the activated CD8α^+^ cDCs (CD11c^int^ I-A^hi^) for at least 24 hr. CD8α^+^ cDCs are known to produce more IL-12 than CD4^+^ cDCs [37]. BCL6^−/−^ DCs, compared to WT DCs, were shown to generate less IL-12 and higher amounts of IL-6 upon LPS stimulation [14]. However, alum injection, which has less adjuvanticity, did not lead to the phenotypic change of CD8α^+^ cDCs (expression of CD11c and MHC II, data not shown), and reduction of BCL6 expression was less profound than was observed with LPS injection (Fig. S2). Therefore, it is tempting to speculate that the differential regulation of BCL6 in CD8α^+^ splenic cDCs may influence the production of inflammatory cytokines.

Using MyD88^−/−^ TRIF^−/−^ mice, we found that LPS-induced transient separation of CD11c^int^ I-A^hi^ subpopulation and reduced BCL6 levels in CD11c^int^ I-A^hi^ cDCs are dependent on MyD88 and TRIF-mediated signaling. A previous study showed that both MyD88 and TRIF pathways are required for LPS-induced DC maturation and up-regulation of costimulatory molecules [38]. Following TLR4 aggregation upon LPS binding, both MyD88-dependent and -independent pathways induce the activation of NF-κB [32]. In GC B cells, CD40 signaling leads to NF-κB activation and subsequent induction of the transcription factor IRF4, which in turn represses BCL6 transcription [39]. It would be of interest to determine if NF-κB activation contributes to the reduction of BCL6 levels in matured/activated cDCs as well.

The expression patterns of Zbtb46, another BTB-Zinc finger transcription factor, is similar in some regards to that of BCL6 in DCs. Similar to BCL6, Zbtb46 expression in DCs is restricted to pre-cDCs and cDCs [40], [41]. TLR stimulation also leads to rapid downregulation of Zbtb46 in cDCs [42]. Moreover, Zbtb46-deficient cDCs appear phenotypically more activated under steady state conditions [42], consistent with our flow cytometry and histology data demonstrating that a reduction of BCL6 protein levels in cDCs is coincident with upregulation of maturation markers.

In summary, BCL6 is not required for the transition from CDPs to pre-cDCs, or the expansion of pre-cDCs within BM, but is coincident with the differentiation of pre-cDCs towards cDCs in peripheral lymphoid organs. BCL6 protein is expressed in cDCs under steady state conditions, especially in the CD8α^+^ subset, but not in pDCs. Upon inflammation, BCL6 is rapidly downregulated within the CD11c^int^ I-A^hi^ subpopulation of cDCs in secondary lymphoid organs, particularly the CD8α^+^ subset, a shift that is reliant on the MyD88 and TRIF pathways. Finally, higher BCL6 levels correlate with greater expression of the proliferation marker Ki-67 in cDCs in the steady state and inflammatory conditions.

## Supporting Information

Figure S1 Alum-induced splenic CD11c^int^ I-A^hi^ cells does not contain monocyte-derived DCs. C57BL/6 mice were injected with LPS or alum by *i.p.* route. 2 days after alum injection *(A)* or 1 day after LPS injection *(B)*, splenocytes were isolated and surface stained with DC markers and monocyte markers, and then analyzed by flow cytometry. Representative plots show that 2 days after alum injection, few Ly6c^+^CD14^+^ cells were observed in either CD11c^hi^ I-A^int^ or CD11c^int^ I-A^hi^ populations *(A)* and that by 1 day after LPS injection, very few Ly6c^+^CD14^+^ cells observed in CD11c^int^ I-A^hi^ cells *(B)*.
(TIFF)

Figure S2 Modulation of BCL6 expression in DC subsets upon LPS/alum injection. C57BL/6 mice were injected with LPS or alum by *i.p.* route. Splenocytes were isolated before (D0) and after (D1, D2) injection, and the BCL6 expression patterns assessed among CD4^+^, CD8α^+^ and CD4^−^CD8α^−^ cDC subsets. *(A)* Average BCL6 MFI ±SEM (n = 4) in cDC subsets within total splenic cDCs. *(B)* Average BCL6 MFI ±SEM (n = 4) within CD11c^hi^ I-A^int^ cells in cDC subsets. *(C)* The average BCL6 MFI ±SEM (n = 4) within CD11c^int^ I-A^hi^ cells in cDC subsets. The left panels represent LPS injection results, and the right panels alum injection results. *p<0.05; **p<0.005; ***p<0.001 (two way ANOVA) was compared within each group to expression levels of BCL6 at D0.
(TIFF)
