# Supplementary figures and images for: Dynamic Expression of BCL6 in Murine Conventional Dendritic Cells during In Vivo Development and Activation
Source: PLoS One. 2014 Jun 30;9(6):e101208. doi: 10.1371/journal.pone.0101208 (PMC4076320; doi:10.1371/journal.pone.0101208)

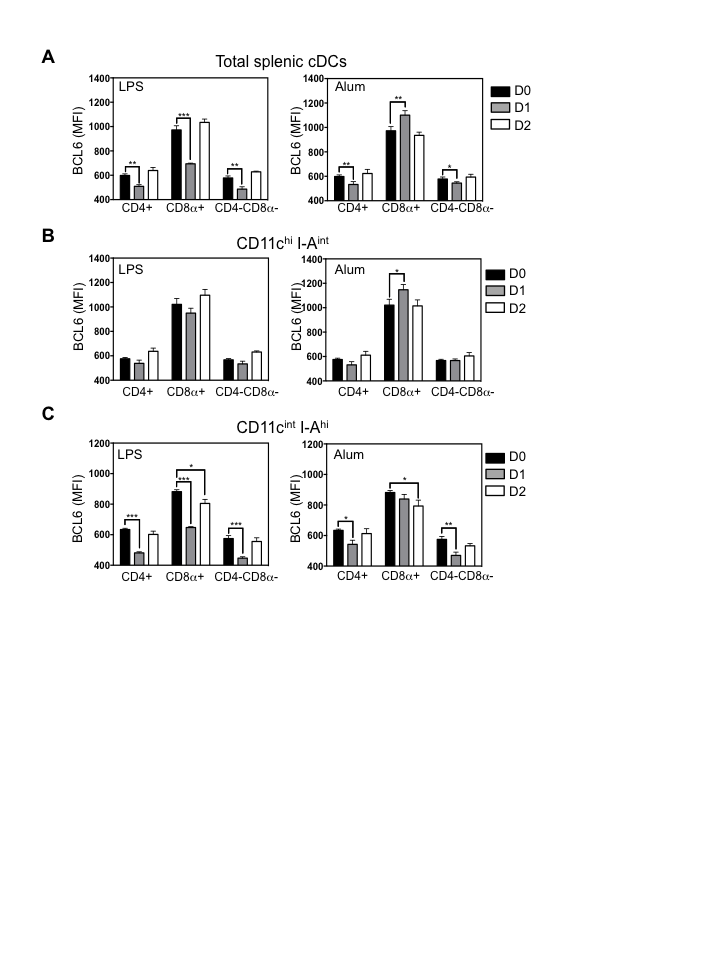

Supplement: Figure S2 — Modulation of BCL6 expression in DC subsets upon LPS/alum injection. C57BL/6 mice were injected with LPS or alum by i.p. route. Splenocytes were isolated before (D0) and after (D1, D2) injection, and the BCL6 expression patterns assessed among CD4+, CD8α+ and CD4−CD8α− cDC subsets. (A) Average BCL6 MFI ±SEM (n = 4) in cDC subsets within total splenic cDCs. (B) Average BCL6 MFI ±SEM (n = 4) within CD11chi I-Aint cells in cDC subsets. (C) The average BCL6 MFI ±SEM (n = 4) within CD11cint I-Ahi cells in cDC subsets. The left panels represent LPS injection results, and the right panels alum injection results. *p<0.05; **p<0.005; ***p<0.001 (two way ANOVA) was compared within each group to expression levels of BCL6 at D0. (TIFF) [file pone.0101208.s002.tiff]
